# Factors that influence performance in Olympic air-rifle and small-bore shooting: A systematic review

**DOI:** 10.1371/journal.pone.0247353

**Published:** 2021-03-31

**Authors:** Sina Spancken, Hannah Steingrebe, Thorsten Stein

**Affiliations:** BioMotion Center, Institute of Sports and Sports Science, Karlsruhe Institute of Technology, Karlsruhe, Germany; University of Illinois at Urbana-Champaign, UNITED STATES

## Abstract

**Background:**

Air-rifle and small-bore shooting are fascinating Olympic sports due to their unique performance requirements for accuracy and precision.

**Objective:**

The purpose of our study was to systematically research the literature to determine and summarize performance determinants in both air-rifle and small-bore shooting. Since some athletes participate internationally in both disciplines in competition, the disciplines must have some similarity in the performance structure. Therefore, we further investigated whether performance in air-rifle and small-bore shooting can be explained by the same performance determinants.

**Methods:**

We systematically searched in four databases using combined keywords relevant to performance in air-rifle and small-bore shooting. The articles included had undergone peer-review and had a) a direct relation to shooting performance, b) an indirect relation by comparing the performance of shooters of different skill levels, and c) a practical relevance (directly controllable through training). After the quality of each article was assessed, the key data were extracted and summarized.

**Results:**

The fourteen articles included achieved an average of 60 ± 14% (range 30–80%) in quality assessment. Altogether, articles covered 268 subjects (32% female), of which 19% were elite- and 28% were national-level athletes. Sixteen performance determinants were investigated, which were divided into anthropometric, technical-coordinative, physiological and psychological categories. Both in air-rifle and small-bore shooting, rifle stability and body sway were found to differ between elite- and national-level athletes. In both disciplines, body sway seemed to have no influence on shot score in elite- and national-level athletes. Similarly, heart rate did not correlate with shot score at nearly all performance levels in both disciplines. In national-level air-rifle athletes, horizontal rifle stability, aiming accuracy and aiming time were found to affect shot score.

**Conclusions:**

To be competitive at a national-level in air-rifle shooting, a highly developed aiming process is needed to achieve a high shot score. Lack of data prevented us from drawing evidence-based conclusions in elite-level air-rifle athletes and in small-bore shooting. Future research should investigate possible performance determinants both in air-rifle and in small-bore shooting, especially with elite-level athletes, to confirm or disprove existing findings. Further research should use more complex analyses to investigate the multifaceted processes associated with different performance determinants.

## Introduction

Shooting is an accuracy and precision sport that can be performed by any individual irrespective of age, sex and performance level [1]. The sport is very popular and is represented at the Olympic Games with 15 disciplines. Of these, air-rifle and small-bore shooting have been part of the Olympic program since 1984 and form the focus of this article.

In air-rifle shooting, 60 shots are fired in the free-standing position and must be completed within 75 minutes. The target, which is 10 m away from the shooter, has a “ten” ring with a diameter of 0.5 mm and lower rings which follow each at a distance of 2.5 mm. In small-bore shooting, 120 shots are fired in three different positions: kneeling, prone and standing (40 shots each) and must be completed within 165 minutes. The target is 50 m away from the shooter and has a “ten” ring of 10.4 mm in diameter and lower rings that follow at 8.0 mm. In both disciplines, the rings are divided into decimal places, so that a maximum of 10.9 points can be scored per shot [2]. In addition, since 2018, both men and women fire the same number of shots in competition both in air-rifle shooting (women previously had 40 shots) and small-bore shooting (women previously had 3x20 shots). As the descriptions show, the disciplines differ in terms of a) their distance to the target, b) the diameter of the target and the “ten”-ring, c) the number of shots fired, d) the shooting positions, and e) the rifle and ammunition. Nevertheless, in practice, shooters compete internationally in both disciplines on a high level, which means that the performance structure of the two disciplines must have some similarity [3]. For this reason, the present systematic review examines both disciplines.

When looking at average shot scores from the 2019 European Games, it becomes evident that the average shot score for entering the finals in small-bore shooting was with 9.76 points for men and 9.68 points for women lower compared to air-rifle shooting, where at least 10.43 points per shot for women and 10.46 points per shot for men had to be scored to reach the final [3]. This shows the higher level of difficulty of small-bore shooting compared to air-rifle shooting, which can be explained i.a. by the stronger recoil of the small-bore rifle and the different shooting positions, resulting in a higher technical demand. Results also showed that there was little variation in shot scores between men and women, but this difference was not significant, which has been confirmed previously by various studies in air-rifle and small-bore shooting [4–8]. This finding is of scientific relevance as it allows men and women to be considered as one group and to omit gender as a covariate which can lead to increased statistical power by increasing the number of subjects especially in the small collective of elite-level shooters.

Especially in air-rifle shooting, it is clear that fault tolerance is minimal. It is necessary to hit the “ten” ring of the target with every shot, and even a small mistake can ruin the entire competition because every shot counts towards the overall result. A study by Zatsiorsky & Akov [9] into air-rifle shooting showed that at shot release, the angular movement of the weapon must be less than 0.016° to fire a ten-point shot. Accordingly, a high and stable development [10] of the technical elements are necessary to achieve a high precision resulting in a high maximum shot score to be competitive on an international level [1, 5, 11–15]. Therefore, mental skills as well as the connection between postural balance and rifle movement seem to be insightful factors to consider in performance diagnostics.

A study using augmented feedback on postural balance and rifle stability [16] led to improvements in shot score in novice air-rifle shooters, whereas balance training (alone vs. in combination with breathing training) did not improve the score [15]. Although the technical elements must be well-developed, other factors can also influence shooting performance. However, meditation training showed no improvement in shooting performance at post-test in elite-level rifle shooters, although a long-term effect was seen: the seasonal competition performance of the intervention group was significantly better than the control group without meditation training [8]. Such intervention studies are important, but do not provide an overview of the performance structure, i.e. they cannot uncover possible performance determinants. Such knowledge, however, is needed for a better understanding of performance determinants crucial to becoming an expert in shooting. This knowledge is therefore of practical relevance for coaches and athletes, as training can be analyzed and performed more consciously and comprehensively. Moreover, intervention studies could then be developed specifically for these determinants.

To the best of our knowledge, there is no systematic review analyzing performance determinants in Olympic air-rifle and small-bore shooting. Therefore, the major aim of this study is to analyze the current state of research on performance-determining factors in both disciplines. A second aim is to investigate whether performance in air-rifle and small-bore shooting can be explained by the same performance determinants, since the disciplines seem to be very similar.

## Methods

This systematic review is based on the structure and reporting guidelines of PRIMSA (Preferred Reporting Items for Systematic Reviews and Meta Analyses; S1 Table) [17]. The protocol was not registered prior to the initiation of the project.

### Search strategy

The databases Pubmed and SciVerse Scopus, as well as the German high-performance databases BiSP-surf and Sponet were systematically searched on January 14^th^ 2020 using the following term: [("air rifle" OR "small bore" OR “rifle shoot*”) AND (perform* OR shoot* OR shot OR scor*) NOT (catheter* OR tube* OR needle* OR injury OR drain* OR engine)]. The all-field-function was used except in the Pubmed database, where the search string was only used for the title and abstract. In addition, the reference lists of all included articles were searched manually, as well as publications of key researchers (e.g., S. Ihalainen and N. Konttinen).

### Study selection process

**Fig 1:**
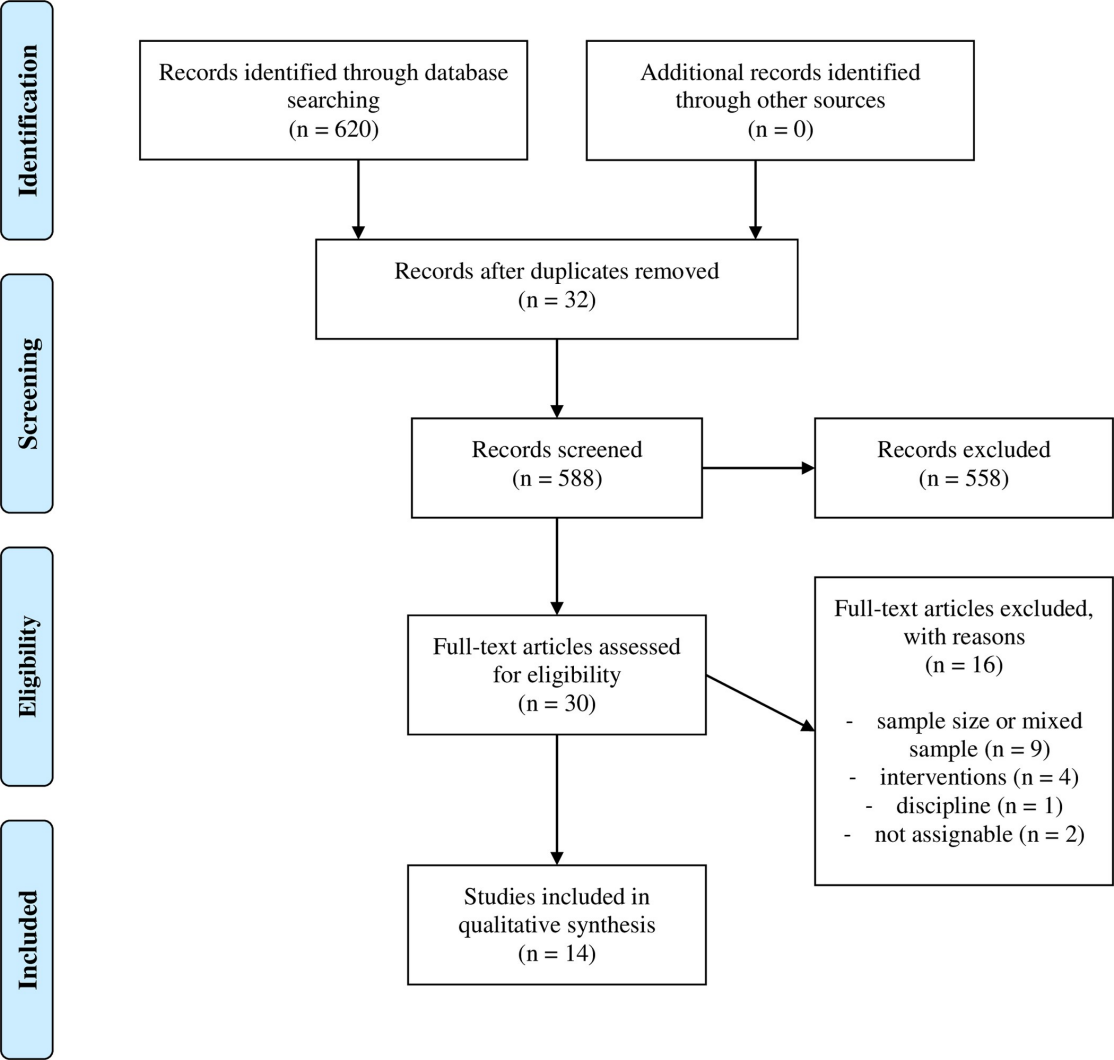
Flow diagram of the search strategy and the article selection process.

First, all duplicate articles within the database were removed. Two independent reviewers (SS and HS) subsequently checked all titles, abstracts and full texts (in this order) for the inclusion and exclusion criteria. Next, the results of the two independent examinations were compared and, where opinions differed, a third reviewer (TS) was consulted. The selection process was repeated for articles identified by manual research strategies (see Fig 1).

### Inclusion and exclusion criteria

Only original research articles published in English from peer-reviewed journals and peer-reviewed conference papers were included. Other types of articles, such as letters to the editor, symposium reports, conference abstracts, technical publications, expert opinions, commentaries, and literature reviews were also excluded. Performance determinants should either be analyzed using direct relation to shot score or indirectly through differentiation between athletes of different skill levels. Studies that examined a different shooting discipline (e.g., pistol or running target) or a rifle other than an air-rifle or small-bore caliber were excluded. In addition, studies were excluded if no clear assignment to air-rifle or small-bore shooting was made, i.e., if neither a description of the sample (e.g., air-rifle athletes) nor the rifle type (e.g., small-bore rifle) or the international competition standards (e.g., 60 shots from a 10m distance) were named. Studies were also excluded that examined mixed samples (e.g., air-rifle and air-pistol shooters), in which no clear separation was made between the disciplines regarding the direct or indirect relation to shot score. Studies with a sample size below five or studies that examined impaired subjects (e.g., visual impairment) were also excluded. Studies using intervention programs or that investigated performance determinants that cannot be controlled by training (not trainable; e.g., electroencephalography (EEG), drugs or nutrition) were excluded.

### Study appraisal

For quality assessment of the included articles, we used a modified version of the Downs and Black Quality Assessment Checklist [18] (S2 Table). It is suitable for different study designs and studies on elite sport performance [19, 20].The original checklist shows high internal consistency (Kuder-Richardson 20 = 0.89), test-retest reliability (r = 0.88), inter-rater reliability (r = 0.75), and criterion validity (r = 0.90) [18].

Several modifications had to be made as the checklist was originally developed for health care interventions. Since intervention studies were excluded, ten questions (number 9, 13–15, 17, 19, 21, 23, 24, 26) could not be answered. For questions 5 and 25, age, skill-level, mean shot score and training and competition experience were defined as covariates. Two points were given when all covariates were reported, one point was given when two or three covariates were reported, and zero points were given when only one or no covariate was reported. For question 27, two points were given when either an *a priori* sample size calculation or a *post-hoc* power analysis was reported, and the power was sufficient; one point was given when an analysis was reported; and zero points were given if no power analysis was reported. We further changed the word “patient” to subject”.

The quality score was calculated as follows: (achieved points / total points of points) x 100%, with a maximum of 18 points. Therefore, higher scores indicate better study quality. Quality assessment for all included articles was made by SS and HS, and again a third reviewer (TS) was consulted in case of discrepancies. All articles were included independently of their quality score.

### Data extraction, synthesis, and analysis

Data concerning the study aims, subjects, experimental protocol, examined parameters and major findings were extracted and analyzed using Microsoft Excel Professional Plus 2019 (Microsoft Corporation, Redmond, Washington, USA). For literature search and administration Citavi 6 (Swiss Academic Software, Zürich, Switzerland) was used. Data extraction was done by one reviewer (SS).

## Results

620 articles were extracted from the databases and 588 articles remained after duplicate removal. When titles, abstracts and full texts were screened, 14 articles that met the inclusion criteria remained. Ten articles were found for air-rifle shooting and four for small-bore shooting. Table 1 summarizes the quality scores, sample characteristics, experimental protocol, examined parameters and key results of each article.

**Table 1:** Summary of the included articles (N = 14) with an overview of the quality scores, sample characteristics, experimental protocols, examined parameters and major results.

Study reference, discipline, quality and focus	Subjects	Experimental protocol	Parameters examined	Major results
Ball et al. (2003) Air-rifle 68% Technic-coordination	Sample: 6 Sex: 4 men + 2 women Age: 22,2 ± 2,6 yrs Skill: Elite Experience: -Shot score: 10.1 ± 0.3	Test: 10m, 20 shots, standing Conditions: AMTI force plates; SCATT system	Shot score Rifle stability Aiming accuracy COP length and range in ML- and AP-direction [mm] 5-0s, 3-0s, 1-0s before shot release	Relationship between body sway and shot score on an intra-individual level but not on an inter-individual level Relationship between body sway and rifle stability on an intra-individual level but not on inter-individual level
Era et al. (1996) Small-bore 36% Technic-coordination	Sample: 24 Sex: 21 men + 3 women Age: 30.1 ± 5.7 yrs Skill: Elite (9) + Nat (8) + + Novices (7) Experience: Elite (10–16 yrs), Nat (12 yrs) Shot score: -	Test: 18m, 100–200 shots dry-firing, standing Conditions: Kistler force plates; Noptel system	Shot score COF velocity, maximum amplitude, moment of velocity in AP- and ML-direction in 1.5s windows, starting 7.5-5s before shot release	Body sway differentiated between skill levels No relationship between body sway and shot score in elite- and national-level shooters Body sway correlated with shot score in novices
Ihalainen et al. (2016) Air-rifle 56% Technic-coordination	Sample: 40 Sex: 18 men + 22 women Age: - Skill: Elite (19)+ Nat (21) Experience: - Shot scores: -	Test: 10m, 40/60 shots, standing 319 analyzed tests with 13797 shots over a period of five years Conditions: Good balance force plates, Noptel system	Shot score Rifle stability, aiming accuracy, cleanness of triggering, aiming time, timing of triggering COP location in ML- and AP-direction 7-2s, 2-0s, 1-0s before shot release	Rifle stability, aiming accuracy, cleanness of triggering and body sway (ML) 7-2s and 1-0s before shot release differentiated between skill levels Rifle stability, aiming accuracy, cleanness of triggering and aiming time correlated with the mean shot score in elite- and national-level athletes Timing of triggering did not correlate with shot score Body sway (AP) correlated with the mean shot score 1-0s before shot release in elite-level athletes Body sway (AP) correlated with horizontal rifle stability 2-0s, 1-0s; Body sway (ML) correlated with vertical rifle stability in all time intervals; Body sway (AP) correlated with horizontal rifle stability 1-0s Regression analysis: Holding stability, aiming accuracy, cleanness of triggering and timing of triggering explained 81% of variance of the shooting score–holding stability explained 54% of variance
Ihalainen et al. (2016b) Air-rifle 66% Technic-coordination	Sample: 17 Sex: 8 men + 9 women Age: 25 ± 6 yrs Skill: Elite Experience: 14 ± 5 yrs Shot score: 10.25 ± 0.14; 10.30 ± 0.08; 10.30 ± 0.07 (season 1–3)	Tests: 10m, 40/60 shots, standing in sim. comp. 15 ± 7 competitions in three years and 11433 analyzed shots Conditions: Good balance force plates, Noptel system	Shot score Rifle stability, aiming accuracy, cleanness of triggering, aiming time, timing of triggering COP location in ML- and AP-direction 7-2s, 2-0s, 1-0s before shot release	Horizontal rifle stability, aiming accuracy and cleanness of triggering correlated with the mean and maximum competition shot score; timing of triggering did not Body sway (ML) correlated with mean and maximum competition shot score during all time intervals before shot release Body sway (AP) correlated with the mean and maximum shot score 1-0s before shot release Body sway (ML+AP) correlated with horizontal rifle stability during all time intervals before shot release Body sway (ML+AP) correlated with cleanness of triggering during all time intervals; except for 7-2s in AP
Ihalainen et al. (2017) Air-rifle 67% Technic-coordination	Sample: 13 Sex: 5 men + 8 women Age: - Skill: Nat (10) + Junior (3) Experience: - Shot score: 10.31 ± 0.13 vs. 10.14 ± 0.17 (training vs. competition)	Tests: 10m, 40 shots, standing Conditions: Good balance force plates, Noptel system	Shot score Rifle stability, aiming accuracy, cleanness of triggering, timing of triggering COP location in ML- and AP-direction 7-2s, 2-0s, 1-0s before shot release	Horizontal rifle stability correlated with shot score in training but not in competition Aiming accuracy was related to shot score in training and competition Vertical rifle stability, cleanness of triggering, timing of triggering and postural balance (AP + ML) in all time intervals were not correlated with the mean training and competition shot score Mean shot score, horizontal and vertical rifle stability, aiming accuracy, cleanness of triggering, body sway (ML) in all time intervals and body sway (AP) 1-0s performance differ between training and competition Absolute change in horizontal rifle stability, aiming accuracy and cleanness of triggering correlated with the absolute change of the mean shot score from training to competition
Konttinen & Lyttinen (1992) Small-bore 30% Physiology	Sample: 6 Sex: 5 men + 1 woman Age: 23–44 yrs (nat); 24–41 yrs (novices) Skill: Nat + Novices (3+3) Experience: - Shot score: -	Tests: 18m, 2x300 shots, standing Conditions: ECG	Shot score Heart rate Breathing pattern	Heart rate and breathing pattern did not correlate with shot score Heart rate and breathing pattern did not differentiate between skill levels
Konttinen et al. (1998) Small-bore 57% Technic-coordination, Physiology	Sample: 12 Sex: 12 men Age: Elite: 32.3 yrs (26–44), Nat: 30.2 yrs (21–34) Skill: Elite + Nat (6+6) Experience: 15.8 (Elite), 13.3 (Nat) Shot score: -	Tests: 10m, 200 shots (100 dry-firing), standing, 18m Conditions: Noptel system, ECG, EMG	Shot score Rifle stability Location of triggering Muscle activity of the trapezius deltoid, brachioradialis and palmaris longus muscles	Heart rate and rifle stability differentiated between skill levels Heart rate patterns were not related to shot score Heart did not correlate with shot score Upper body muscle activity and heart rate correlated with rifle stability
Konttinen et al. (2003) Air-rifle 60% Technic-coordination, Physiology	Sample: 20 Sex: 20 men Age: 26.8 ± 2.5 yrs Skill: Novices Experience: - Shot score: -	Tests: 10m, 200 shots (100 dry-firing), standing Conditions: Noptel system, ECG	Shot score Location of triggering	Shooters prefer triggering in the systolic phase of the cardiac cycle Location of triggering was related to shooting score
Kuitunen et al. (2013) Air-rifle 50% Technic-coordination	Sample: 15 Sex: 5 men + 10 women Age: - Skill: Elite (4) + Nat (11) Experience: - Shot score: -	Tests: 10m, 40/60 shots, standing, elite: 30 tests, national: 28 tests Conditions: Noptel system, force plate (Metitur)	Shot score Rifle stability, aiming accuracy, cleanness of triggering, SD of COP in ML- and AP-direction 7-2s, 2-0s, 0.5-0s before shot release	Horizontal and vertical rifle stability, aiming accuracy, aiming time, cleanness of triggering and body sway (ML) 7-2s, 0.5-0s before shot release differentiated between skill levels Horizontal and vertical rifle stability, aiming accuracy, aiming time and cleanness of triggering correlated with the mean shot score in national-level but not in elite-level athletes Body sway (ML+AP) correlated with the mean shot score in all time intervals in national-level but not in elite-level shooters
Landers et al. (1985) Small-bore 61% Psychology	Sample: 20 Sex: - Age: 18–25 yrs Skill: Nat (10) + Novices (10) Experience: - Shot score: -	Tests: 50m, 25 shots, sitting Conditions: ECG	Shot score Heart rate	Stress level (heart rate) was correlated with shot score
Mets et al. (2007) Air-rifle 57% Technic-coordination, Physiology	Sample: 20 Sex: 15 men + 5 women Age:16.1 ± 2.1 yrs Skill: Junior Experience: 5.9 ± 2.0 yrs Shot score: -	Tests: 10m, 200 shots dry-firing, standing Retention: 4-weeks Conditions: Noptel system, ECG	Shot score RR-interval length Location of triggering	Shooters prefer triggering in the systolic phase of the cardiac cycle Location of triggering and heart rate did not correlate with shot score Location of triggering did not correlate with heart rate
Mon et al. (2019) Air rifle 67% Anthropometry	Sample: 14 Sex: men + women Age: - Skill: Junior Experience: 46,75±20,92 months (male), 52,00±24,79 months (females) Shot score: 9.05 ± 0.62 (men); 9.47 ± 0.16 (women)	Tests: 10m, 40/60 shots, standing, competition Conditions: Kistler force plate	Shot score COP (displacement, velocity) in ML- and AP-direction	Body (ML + AP) sway did not correlate with mean shot score
Sade et al. (1990) Air-rifle 73% Psychology	Sample: 55 Sex: 37 men + 18 women Age: 28.7 ± 7.2 Skill: Elite (28), Nat (27) Experience: - Shot score: rounded over 7 competitions: Elite: 545.01 ± 15.41; Nat: 341.76 ± 16,78	Tests: 10m, 60 shots, standing, across 7 competitions Conditions: anxiety and self-control questionnaire	Shot score Anxiety and self-control score	Trait anxiety and self-control did not differentiate between skill level, but state anxiety did Trait anxiety and self-control correlated weakly with shot score State anxiety correlated with shot score
Selva & Jopseh (2017) Air-rifle 39% Technic-coordination	Sample: 6 Sex: 4 men+ 2 women Age: - Skill: Nat Experience: - Shot score: -	Tests: 10m, 40/60 shots, standing Conditions: pressure insoles	Shot score Body sway in ML- and AP-direction (SD of displacement velocity) Body weight distribution of left and right foot	Body weight distribution and body sway in ML- and AP-direction did not correlate with shot score

Yrs = years, COP = center of pressure, ML = medio-lateral, AP = anterior-posterior, Nat = National, COF = center of force, ECG = electrocardiogram, EMG = electromyogram, SD = standard deviation, RR = R wave to R wave.

### Quality score

The average quality score of the 14 articles was 56 ± 13% (range 30–73%). Most quality points were lost due to lack of subject data (i.e., representativeness, recruitment, and inclusion criteria), lack of control for possible covariates, lack of precise reporting of significance level, lack of power analyses, and lack of reliability/validity data of the measuring instruments.

### Subjects and experimental protocols

In total, 268 subjects were included, 77% in air-rifle and 23% in small-bore shooting. The mean sample size was 19 ± 14 subjects (range 6–55) per study. Most subjects (60%) were male, 32% were female and for one study sex was not specified. The mean age of subjects across the studies was 23.8 ± 4.1 years, but five studies did not mention the age of their subjects and five studies reported only the mean age or the age range without specifying the dispersion. Of the 268 athletes, 19% were ranked as international or elite-level athletes, 28% were ranked as national-level athletes, 14% were junior-national or juvenile athletes, 15% were inexperienced or novice and 24% were ranked experienced or highly and moderately skilled.

Fifty percent of the studies reported the mean shot score of the investigated athletes. The mean shot score for elite-level air-rifle athletes in simulated competitions was 10.27 ± 0.15 (three studies). National-level air-rifle athletes scored on average 10.25 ± 0.1 points per shot in simulated competitions (four studies) and 9.94 ± 0.23 in competition (two studies). There was one study in junior-level air-rifle athletes (9.26 ± 0.39 points per shot), as well as for novice (7.25 ± 2.22), highly skilled (9.08 ± 0.9) and moderately skilled (5.70 ± 0.28) air-rifle shooters. Studies in small-bore shooting did not report any mean shot scores.

In air-rifle shooting, 10 studies tested performance according to international standards, e.g., 10 m away from the target, whereas in small-bore shooting four studies reduced the original 50 m distance to 18 m and 15,24 m, respectively. All shooters shot in the standing position, except for one study in small-bore shooting which chose a sitting position. Seven studies in air-rifle shooting shot 40 and/or 60 shots (women/men) according to international standards, two studies reduced the number to 20 and 25 shots, and five studies had a much higher number of shots (between 100 and 300). Across all studies, one study investigated shooting performance in competition, nine studies investigated shooting performance in training, three studies examined shooting performance both in training and competition and one study did not assess shooting performance. Eight studies used force plates or pressure insoles to assess shooters’ body sway and nine studies used optoelectronic systems to record the aim-point trajectory of the rifle. Moreover, electrocardiography (five studies), electromyography (one study), or questionnaires (two studies) were used.

### Performance factors

In the included studies a total of 16 performance determinants were investigated, of which 13 were in air-rifle and six were in small-bore shooting. To enhance the comprehensibility and readability of the review we divided these performance determinants into four different categories: a) anthropometric, b) technical-coordinative, c) physiological, and d) psychological performance. For small-bore shooting, we could only analyze standing shooting, even though it’s a three-position discipline, except for one study, who investigated shooting performance in a sitting position. The resulting performance determinants were partly not congruent between the two disciplines (see Table 2 for details).

**Table 2:** Investigated performance determinants in air-rifle and small-bore shooting (performance determinants are described in the results section).

	Air-rifle shooting	Small-bore shooting
Anthropometric Body weight Body height	Mon et al. (2019) Mon et al. (2019)	
Technical-coordinative Horizontal rifle stability Vertical rifle stability Aiming accuracy Cleanness of triggering Timing of triggering Aiming time Body sway Load distribution between feet	Ihalainen et al. (2016; 2016b; 2017), Kuitunen et al. (2013) Ihalainen et al. (2016; 2017), Kuitunen et al. (2013) Ihalainen et al. (2016; 2016b; 2017), Kuitunen et al. (2013) Ihalainen et al. (2016; 2016b; 2017), Kuitunen et al. (2013) Ihalainen et al. (2016; 2016b; 2017) Ihalainen et al. (2016), Kuitunen et al. (2013) Ball et al., (2003), Ihalainen et al. (2016; 2016b; 2017), Kuitunen et al. (2013), Mon et al. (2019) Selva & Joseph (2017)	Konttinen et al. (1998) Konttinen et al. (1998) Era et al. (1996)
Physiological Location of triggering Heart rate Upper body muscle activity Breathing pattern	Konttinen et al. (2003), Mets et al. (2007) Konttinen et al. (2003), Mets et al. (2007)	Konttinen et al. (1998), Konttinen & Lyttinen (1992) Konttinen & Lyttinen (1992) Konttinen & Lyttinen (1992)
Psychological Trait and state anxiety Self-control Stress	Sade et al. (1990) Sade et al. (1990)	Landers et al. (1985)

#### Anthropometric parameters

One study investigated the effects of anthropometric measurements on shot performance in juvenile air-rifle shooters, and found that neither body weight nor height seemed to affect shot performance [21].

#### Technical-coordinative parameters

Nine papers looked at technical-coordinative factors in air-rifle (seven studies) and small-bore (two studies) shooting, investigating seven different performance determinants regarding postural balance and aim point trajectory of the rifle (Table 2). Postural balance was assessed as body sway using the fluctuation of the center of pressure (COP) [5, 11, 12, 22, 23] in different time intervals before shot release, which ranged from 7.5s to 0.5s before triggering the shot. In addition, all studies divided body sway into a medio-lateral component (ML; movement in shooting direction) and an anterior-posterior component (AP; movement perpendicular to shooting direction). Aim point trajectory was described through six parameters with different operationalization. Rifle stability in the horizontal and vertical direction was calculated as the standard deviation (SD) of the aim point location during the last second before shot release [5, 22–24]. Aiming accuracy was either described as the mean location of the aiming point during the last second [5, 22, 23]; or the time that the aim point spent in the 10-ring during the last second before shot release as a percentage of aiming time [24]. Cleanness of triggering was measured as the movement of the aiming point during the last 0.2 s before shot release [5, 22–24]. Timing of triggering was the time period when the mean location of the aiming point is closest to the center of the target [5, 22, 23]. Aiming time was defined as the time, the aim point was continuously located on the target [5, 24].

*Air-rifle shooting*. Studies comparing groups found a difference between elite- and national-level air-rifle athletes concerning horizontal and vertical rifle stability, aiming accuracy and cleanness of triggering [5, 24], i.e., that elite-level athletes showed better values for horizontal and vertical rifle stability, aiming accuracy and cleanness of triggering compared to national-level athletes. Timing of triggering did not differ between elite- and national-level athletes [5] and for aiming time, inconsistent results were found. Kuitunen et al. [24] found longer aiming times for elite-level athletes compared to national-level athletes, while Ihalainen et al. [5] did not find a difference.

In elite-level air-rifle athletes, inconsistent results were found for the correlation of horizontal and vertical rifle stability, aiming accuracy, cleanness of triggering, and aiming time with mean shot score. Here again, this was because one study found a correlation [5] whereas another did not [24]. Timing of triggering showed no correlation with mean shot score in either national- [5, 23] or elite-level air-rifle athletes [5]. Further, in national-level air-rifle athletes, horizontal rifle stability, aiming accuracy [5, 23, 24], and aiming time [5, 24] all correlated with mean shot score. However, inconsistent results were found for vertical rifle stability and cleanness of triggering, which was because two studies found a correlation with the shot score [5, 24] whereas the other did not [23].

Body sway in the ML-direction 7–2 s, 1–0 s, and 0.5–0 s before shot release [5, 24] was different between elite- and national-level air-rifle athletes, as well as for body sway in the AP-direction 2–0 s and 1–0 s [5] before shot release with elite-level athletes showing lower body fluctuations compared to national-level shooters.

In elite-level air-rifle athletes, no correlation was found between mean shot score and body sway in both directions during several time intervals before shot release [5, 11, 24]; except at 1–0 s in AP-direction [5]. In national-level athletes, inconsistent results were found in the relationship of body sway and mean shot score at several time intervals before shot release (7–2 s; 2–0 s; 1–0 s and 0.5–0 s). This is because one study [24] found a relationship between shot score and body sway in both directions, and three studies did not [5, 23, 25]. Another study by Selva & Joseph [25] with national-level air-rifle shooters showed that body weight distribution on the left and right foot did not correlate with shooting performance.

In addition, correlation analyses were reported between two technical-coordinative performance determinants. In elite-level air-rifle athletes, correlations were found between body sway in the ML- and the AP-directions during all analyzed time intervals before shot release and horizontal rifle stability and cleanness of triggering; except for body sway in the AP-direction 7–2 s before shot release and cleanness of triggering [22]. Contrary results were found by Ball and colleagues in elite air-rifle shooters [11], who found correlations between body sway and rifle stability on an intraindividual level, but not on an interindividual level. When looking at relationships between body sway and rifle stability in a mixed sample of elite- and national-level air-rifle athletes, body sway in the AP-direction 2–0 s and 1–0 s before shot release correlated with horizontal rifle stability [5]. Further, body sway in the AP-direction 1–0 s before shot release correlated with vertical rifle stability and body sway in the ML-direction during all time intervals before shot release showed a correlation with vertical rifle stability [5].

Regression analyses have also been used to investigate rifle stability, aiming accuracy, cleanness of triggering and timing of triggering as key performance determinants in air-rifle shooting. These parameters explained 81% of the variance of the mean shooting score. It was found that rifle stability was the most important factor, explaining 54% of variance [5]. This regression equation was valid for both elite- and national-level athletes and both in simulated and actual competitions [5, 23].

An important point in investigating performance determinants is the consideration of competition performance. Two studies of national-level and juvenile air-rifle athletes investigated the influence of technical-coordinative parameters measured in training, and compared these with shooting performance in competition [21, 22]. Here, horizontal rifle stability, aiming accuracy and cleanness of triggering were correlated with the mean and maximum shot score in competition, whereas timing of triggering did not [5]. Further, body sway in the ML-direction 7–2 s, 2-0s, and 1–0 s before shot release, and in the AP-direction 1–0 s before shot release, were both correlated with mean and maximum competition score in national-level air-rifle shooters [22], but not in juvenile air-rifle athletes [26]. Another study measured technical-coordinative parameters in training and competition [23]. In national-level air-rifle athletes, mean shot score, horizontal and vertical rifle stability, aiming accuracy, cleanness of triggering, body sway in the ML-direction during all time intervals and in the AP-direction 1–0 s before shot release differed between training and competition [23]. Further, only aiming accuracy was correlated with mean competition shot score [23]. In addition, the absolute change in horizontal rifle stability, aiming accuracy and cleanness of triggering correlated with absolute change of mean shot score from training to competition [23].

*Small-bore shooting*. In small-bore shooting, horizontal and vertical rifle stability [13], as well as body sway in both the ML- and AP-directions differed between elite- and national-level small-bore shooters [12]. This means that elite-level small-bore shooters showed less horizontal and vertical rifle movements and lower body sway values in the ML- and AP-direction compared to national-level small-bore shooters. Further, in elite- and national-level small-bore shooters, no difference was found between body sway in the least and most successful shot scores in both the ML- and AP-directions; whereas in novice shooters higher body sway values were reported in poor shots [12].

#### Physiological parameters

Four papers investigated four particular physiological factors in air-rifle and small-bore shooting: heart rate and location of triggering within the cardiac cycle, breathing patterns and upper body muscle activity.

*Air-rifle shooting*. Results showed that novice and junior-level air-rifle shooters preferred triggering at the beginning of the cardiac cycle, that means in the systolic phase [27, 28]. Further, in novice air-rifle shooters location of triggering within a cardiac cycle was related to shot score [27], where triggering at the beginning (0–50%) and at the end (70–99%) of the cardiac cycle led to higher shot scores. In junior-level air-rifle shooters location of triggering showed no correlation with the shot score [28]. In novice and junior-level air-rifle shooters, no correlation was found between heart rate and shot score [27, 28]. In addition, in junior-level air-rifle athletes, no correlation was found between location of triggering and heart rate [28].

*Small-bore shooting*. In small-bore shooting, heart rate differed between elite- and national-level athletes [13], showing higher heart rates for elite-level athletes compared to national-level shooters. However, no difference was found between national-level and novice shooters [29]. Further, elite athletes showed less muscle activity in the upper body than national athletes [13]. Breathing patterns did not differ between national- and junior-level shooters [29]. In elite- and national-level shooters, no correlation was found between heart rate and shot score [13, 27–29]. However, upper body muscle activity showed a correlation with rifle stability in elite- and national-level shooters [13]. Also, in novice and national-level athletes, breathing patterns showed no influence on shooting performance [29].

#### Psychological parameters

Two papers analyzed psychological factors in air-rifle and small-bore shooting: self-control and trait and state anxiety, measured with questionnaires, and stress, measured via heart rate. Trait anxiety describes a person’s tendency to sense anxiety, while state anxiety is perceived as a reaction to a specific situation, e.g., competition. Self-control and trait anxiety were measured a few days before competition while state anxiety was measured immediately before competition.

*Air-rifle shooting*. In air-rifle shooting, highly and moderately skilled athletes did not differ in terms of trait anxiety and self-control, but state anxiety was lower among highly skilled shooters compared to moderately skilled athletes [26]. Trait anxiety and self-control showed very weak positive correlations, state anxiety negative correlation with competition shot score in a mixed sample of elite- and national-level air-rifle shooters, resulting in better shot scores at lower state anxiety levels [26].

*Small-bore shooting*. In small-bore shooting, Landers et al. [30] showed a correlation between stress level and shot score in national-level shooters, resulting in better shot scores at lower stress levels.

## Discussion

The purpose of this study was to systematically search and summarize the literature on performance-determining factors in air-rifle and small-bore shooting. A secondary objective was to compare the two disciplines to determine whether shot performance can be explained by the same performance determinants. Based on our search strategy, we found 14 original research articles that met the inclusion criteria, which indicates that this field has not been extensively researched. Nevertheless, 16 performance factors were investigated and, categorized into anthropometric, technical-coordinative, physiological and psychological factors. Six of them showed a significant correlation with shooting performance, pointing towards a complex and multifactorial character of shooting performance. These correlations were mainly found in national-level air-rifle athletes.

### Anthropometric factors

#### Air-rifle shooting

One study in air-rifle shooting showed that body weight and height did not seem to affect competition shot score of juvenile shooters [21]. These results are in line with national-level pistol shooters [31], suggesting that the corridor for body height and weight is relatively large, unlike in other sports (e.g., gymnastics), where a certain height or weight is required to be successful. However, further studies are needed to confirm this finding to make evidence-based conclusions about not using body weight and height as performance determinants to discriminate between shot scores in juvenile air-rifle shooters or between skill levels and shooting performance in high performance athletes. Other anthropometric data (e.g., segment lengths, ratios between segments, body fat in limbs), hypothesized to be relevant for shooting techniques, should be further investigated. This could also answer the question of whether certain body constitution types lead to higher shooting performance.

### Technical-coordinative factors

#### Air-rifle shooting

In air-rifle shooting, studies comparing groups found that elite-level athletes had better values for horizontal and vertical rifle stability compared to national-level athletes [5, 24, 27]. Studies in elite- vs. national-level rifle shooters [32, 33], as well as in national-level vs. novice pistol shooters [34] evidenced that rifle stability differed between skill levels. Konttinen and colleagues [32] further suggest that the rifle of the elite shooters remained in a stable position for longer compared to national-level athletes, who used the first moment of steadiness for triggering the shot. Results indicate that elite-level air-rifle shooters can take a more stable rifle position before they pull the trigger than national-level athletes, which is why shot score may be determined by a higher holding ability. Therefore, horizontal rifle stability can be used as a performance determinant to discriminate between elite- and national-level air-rifle athletes. Further, in air-rifle shooting, elite-level athletes showed better values for aiming accuracy and cleanness of triggering [5, 24] compared to national-level athletes. This could be interpreted that better aiming and triggering may be critical for better shooting performance; and that these factors may be used as performance determinants to discriminate between elite- and national-level air-rifle athletes. However, no difference was found between timing of triggering in elite- vs. national-level air-rifle shooters [5]. Thus, timing of triggering may not play a role in shooting performance.

In studies within elite-level athletes, no general statement can be made regarding the relationship between aim-point trajectory factors and shot score, since inconsistent results were found. One study found a correlation between shot score and horizontal rifle stability, aiming accuracy, cleanness of triggering, and aiming time [5] but the other did not [24]. Again, no correlation with shot score was found for timing of triggering [5]. These results are somewhat consistent with elite-level rifle athletes, where rifle stability differed between high and low scoring shots [33]; and with elite-level pistol shooters, where a higher pistol stability and a better aiming accuracy led to higher shot scores [35]. Significant correlations may allow the same interpretations as for national-level athletes. In contrast, non-significant relationships could indicate that aim-point trajectory factors may already be at such a high level, resulting in homogeneous performance in elite-level athletes. This results in low variances between athletes and non-significant relationships. That is why several authors [11, 24, 35] advocate using intra-individual analyses for high performance-level athletes in addition to inter-individual analyses. Accordingly, no evidenced-based conclusions can be drawn regarding the influence of aim-point trajectory variables on shot score due to inconsistent results within elite-level athletes and require future research.

In studies within national-level athletes, lower values for horizontal rifle stability (less rifle movement) led to higher shot scores [5, 23, 24]. However, inconsistent results were found for vertical rifle stability, since two studies [5, 24] found a correlation with mean shot score and one study did not [23]. These results are similar to studies of national-level pistol shooters [36], a mixed sample of elite-, national- and junior-level biathletes [37] and conscripts [38], who showed a correlation between horizontal rifle stability and shot score. Results got also confirmed for vertical rifle stability with a mixed sample of national- and junior-level biathletes [39], where a more stable aiming process led to higher shot scores. Furthermore, our results agree with a study of national-level rifle shooters [33], and a mixed sample of elite-, national- and junior-level biathletes [40], who showed a correlation between rifle stability and shot score. Results are also in line with Konttinen et al. [16], who showed that shot score improved when using augmented feedback on rifle stability in novice air-rifle shooters. Furthermore, a higher aiming accuracy was associated with a higher shot score in national-level athletes [5, 23, 24]. This finding is consistent with Hawkins et al. [36, 41], who found this effect in national-level pistol shooters. Results could be interpreted that a better aiming process is critical for better shooting performance. In particular this could mean that the smaller the variance of the location of the aiming point (rifle stability) was and the closer the mean aim point was located to the center of the target (aiming accuracy) during the last second before shot release, the higher the shot score. This could also mean that horizontal rifle stability and aiming accuracy can be used as performance determinants to discriminate between shot scores within national-level air-rifle athletes. Also, vertical rifle stability may be used as a performance determinant, but further analyses are needed due to inconsistent results.

In national-level air-rifle athletes higher aiming times led to higher shot scores [5, 24]. These results do not agree with Hawkins [36], who showed no influence of aiming time on shot score in national-level pistol shooters. However, inconsistent results were found for cleanness of triggering, since two studies found a correlation with the mean shot score [5, 24] and one study did not [23]. These results are in line with Hawkins [36] in national-level pistol shooters and Ihalainen et al. [39] in junior-level biathletes, who showed that lower values for cleanness of triggering led to higher shot scores. However, timing of triggering was independent of shot score [5, 23]. This could mean that an optimal aiming time and triggering process are critical for a better shooting performance. Aiming time may be used as a performance determinant to discriminate between shot scores in national-level air-rifle athletes. Also, cleanness of triggering may be used as a performance determinant, but further analyses are needed due to inconsistent results. However, timing of triggering may not play a role in shooting performance in these athletes and may therefore not be useful as a performance determinant.

In air-rifle shooting, studies comparing groups found that elite-level athletes showed lower values for body sway in both the ML- and AP-directions during several time intervals before shot release compared to national-level athletes [5, 12, 24]. These results are in line with Aalto et al. [42], who showed lower values for body sway in a mixed sample of elite- and national-level rifle and pistol shooters compared to novices; and also with Mon et al. [14], who showed lower values for body sway in a mixed sample of elite-level rifle and pistol shooters compared to national-level athletes. These results also comply with Ko et al. [34], who showed that body sway differed between national-level and novice pistol shooters. Taken together, these results suggest that better postural balance may lead to a higher shot score. Therefore, body sway may be used as a performance determinant to discriminate between elite- and national-level air-rifle athletes.

However, in studies within elite-level shooters, body sway showed no influence on shot score [5, 11, 24]. These results are in line with Ball et al. [35] in elite-level pistol shooters, but in contrast to Sattlecker et al. [40], who showed a correlation between body sway and shot score in biathletes of different skill levels, where lower body fluctuations led to higher shot scores. However, it should be mentioned that Sattlecker et al. [40] did not control for skill level in their analysis and they also considered a different sport in which balance may play a much bigger role: the slim skis are an unstable base, and this could limit the comparability with elite-level air-rifle shooters. Result suggest that elite-level air-rifle athletes may have developed their postural balance so well that body sway may no longer differ between shot scores. Therefore, a low body sway should be considered as a performance requirement to participate at an elite level, rather than as a performance determinant.

In national- and juvenile-level air-rifle shooters, one study found a correlation between body sway and shot score [24] and four studies did not [5, 11, 23, 43]. In contrast, studies in biathletes of different skill levels [40] and conscripts [38] showed a correlation between body sway and shot score. Further, the results comply with Park et al. [15] in novice air-rifle shooters, who showed no improvements in shot score after balance training. Again, it should be mentioned that Sattlecker et al. [40] did not control for skill level in their analysis, which can limit the comparability with national-level air-rifle athletes. In novice athletes, shooting-specific postural balance may not yet be developed, which means that body sway may influence their score. Results suggest that postural balance may already be so well developed among all athletes that body sway no longer influences shot score, which is why a low body sway should be considered as a performance requirement rather than a performance determinant in national-level air-rifle athletes.

In air-rifle shooting, correlation analyses were reported between two technical-coordinative factors. In studies within elite-level athletes, one study found a correlation between body sway and rifle stability [22] and one study did not [11]. Further, in a mixed sample of elite- and national-level air-rifle athletes, a correlation was found between body sway and rifle stability [5]. These results are somewhat in line with studies by Sattlecker et al. [37, 40] in biathletes of different skill levels, which showed correlations between body sway and rifle stability; and also a study by Ball et al. [35] in elite-level pistol shooters, which reported no relationship between body sway and pistol stability. It is both conceivable that low body fluctuations may lead to low rifle movements as well as that body fluctuations may be compensated by opposing rifle movements. It is thus conceivable that the relationship between postural balance and rifle stability plays a role for shooting performance with rifle stability being a mediator. However, further research with separated performance classes is needed to confirm this finding.

An important point in investigating performance determinants is the consideration of competition performance. So far, nearly all studies examined shooting performance and possible performance determinants in simulated competitions during training (partly under laboratory conditions). A study by Ihalainen and colleagues [23] in national-level air-rifle athletes examined technical-coordinative performance determinants both in training and competition. For horizontal and vertical rifle stability, aiming accuracy, cleanness of triggering and body sway in both directions, a performance loss was found from training to competition, which resulted in a worse shot score in competition compared to training [23]. However, only aiming accuracy showed an influence on shot score in competition [23]. When comparing technical-coordinative performance determinants measured in training with actual competition shot scores, horizontal rifle stability, aiming accuracy and cleanness of triggering were correlated with the mean and maximum competition shot score in national-level air-rifle athletes [22]. Further, body sway in both the ML- and AP-directions influenced shot score in national-level air-rifle shooters [22], but not in juvenile athletes [21]. However, Mon and colleagues [21] examined postural balance when barefoot and using a barbell instead of an air-rifle which limits the transferability of the results. The relationship between body sway and shot score indicates that postural balance in competition may be used as a performance determinant rather than a performance requirement since athletes with a lower body sway (especially in the ML-direction) achieved higher shot scores. Further research is crucial to substantiate this result, since in training or simulated competition body sway did not influence shot score. It remains unclear why there was no influence of horizontal rifle stability on shot score in competition. One crucial aspect is that further studies should investigate technical-coordinative factors in competition to find which may be decisive.

Multiple approaches, in the form of multiple regression analyses, have been carried out to gain insight into the importance of aim-point-trajectory factors. Ihalainen and colleagues [5] identified rifle stability, aiming accuracy, cleanness of triggering and timing of triggering as the key performance factors in air-rifle shooting, as they explained 81% of the variance of mean shooting score. Rifle stability was the most important factor, accounting for 54% of the shot score. This regression equation was valid for elite- and national-level air-rifle athletes in training and competition [5, 23]. Similar results were found by Mononen and colleagues [38] in conscripts. These authors integrated body sway into their regression model, and showed that body sway in the ML-direction and vertical rifle stability explained 26% of the variance of shot score. Results indicate an interaction between different aim-point trajectory factors, which in turn influenced shot score. Such an interaction can also increase the importance of variables that, on their own, showed no influence on shot score (e.g., timing of triggering) but contribute to total shooting performance. Future research should also integrate body sway variables into multiple regression analyses to gain further insight into the interaction of technical-coordinative performance determinants, which in turn influence shooting performance.

#### Small-bore shooting

In small-bore shooting, studies that compared groups found that elite-level athletes had better horizontal and vertical rifle stability compared to national-level athletes [13]. These results are in line with those of elite- vs. national-level rifle shooters [33], as well as national-level vs. novice pistol shooters [34], where rifle or pistol stability differed between skill levels. Results may indicate that a higher shot score is determined by a higher holding ability and that rifle stability may be used as a performance determinant to discriminate between elite- and national-level small-bore athletes.

In a mixed sample of novice, elite- and national-level small-bore shooters, rifle stability differed between high, moderate and low scores [13]. These results are in line with elite-level, and national-level rifle shooters [33], where rifle stability differed between high and low scoring shots. Further, results were somewhat similar to those of biathletes of different skill levels [37, 40], elite-level [35] and national-level pistol shooters [36], and conscripts [38], where lower values for rifle or pistol stability lead to higher shot scores. Moreover, results are in line with Konttinen et al. [16] in novice air-rifle athletes, who showed improvements in shot score after using augmented feedback on rifle stability. Results suggest that better rifle stability may lead to a higher shot score in a mixed sample of novice, elite- and national-level small-bore shooters. However, since Konttinen et al. [13] did not control for skill level in their analysis, the relevance of the results is limited to mixed samples, and no evidence-based conclusion can be made about rifle stability being a performance determinant in high-level small-bore shooting.

Other inter-group comparisons found that elite-level small-bore shooters had lower body sway in both directions during several time intervals before shot release compared to national-level shooters [12]. These results comply with Aalto et al. [42], who showed lower body sway in a mixed sample of national-level rifle and pistol shooters compared to novices; and with Mon et al. [14], who showed lower values in a mixed sample of elite-level rifle and pistol shooters compared to national-level shooters. Results are also in line with Ko et al. [34], who showed that body sway differed between national-level and novice pistol shooters. These results suggest that a better postural balance could lead to a higher shot score. However, further research should be conducted to confirm this finding.

In studies within elite-level athletes, body sway did not differ between least and most successful shots. In contrast, when comparing novice small-bore shooters with each other, lower body sway values led to higher shot scores [12]. These results are in line with Ball et al. [35] in elite-level pistol shooters and Mononen et al. [38] in conscripts. In elite-level athletes, body sway may already be well developed, so that it no longer influences shot score, while novices may not yet be able to control their body fluctuations. Again, in elite-level small-bore shooters, body sway should be seen as a performance requirement to compete at elite-level rather than a performance determinant; whereas in novice athletes, body sway may be used as a performance determinant to differentiate between shot scores.

#### Summary

Rifle stability and body sway have been investigated in many studies. In air-rifle shooting, results allow the conclusion that horizontal and vertical rifle stability, as well as aiming accuracy, aiming time and body sway, may be used as performance determinants to discriminate between elite- and national-level athletes. The results of rifle stability and body sway also apply to elite- vs. national-level small-bore shooters. Within the same performance level, results from national-level air-rifle athletes showed that horizontal rifle stability and aiming accuracy can be used as performance determinants to discriminate between shot scores. Results also allow the conclusion that for elite- and national-level air-rifle athletes, as well for national-level small-bore athletes, body sway cannot be used as a performance determinant to discriminate between shot scores. Instead, it should be used as a performance requirement to compete at an elite or a national level. Since little research has been done in small-bore shooting, future research should focus on investigating aim-point trajectory variables to understand their influence on shooting performance. Further work could investigate if performance in small-bore shooting can be explained by the same performance determinants as in air-rifle shooting. However, future research should not only focus on small-bore shooting, but also on air-rifle shooting to confirm the existing findings concerning aim-point trajectory and body fluctuations. Above all, more studies should examine elite-level athletes to investigate which performance determinants can differentiate between shot scores and which parameters serve as contributing factors. Future research should investigate the role of postural balance, e.g., the effect of body sway as a mediator between rifle stability and shooting performance. Studies with full body motion capture could help analyze segment movements more precisely than COP movements.

### Physiological factors

#### Air-rifle shooting

In air-rifle shooting, results from novice and junior-level shooters showed a preference for triggering at the beginning of the cardiac cycle, during systole [27, 28]. These results are somewhat in line with Helin et al. [44] in novice rifle shooters, who showed a random triggering within the cardiac cycle. In contrast, results differ from a study in junior-level biathletes [45], who showed an increased likelihood of triggering the shot in the middle and at the end of the cardiac cycle, during diastole. However, it should be considered that, in biathlon, triggering within the cardiac cycle may play a much higher role due to the preceding cross-country skiing condition, which means that biathletes may already be able to control triggering within the cardiac cycle at a lower skill level. Helin et al. [44] added that champion rifle shooters triggered during diastole almost consistently.

In studies within novice air-rifle athletes, location of triggering within the cardiac cycle was correlated with shot score [27] but no correlation was found in junior-level air-rifle shooters [28]. These results are similar to Gallicchio et al. [45] in junior-level biathletes, and Helin et al. [44] in novices, who both showed a higher shot score when triggering during diastole. Results indicate that location of triggering may affect shot score in novice air-rifle athletes but may be independent of shot score in junior-level air-rifle shooters. We believe that triggering during systole seems to affect the shot due to the jerk that occurs with blood ejection; this movement may be transferred to the weapon. During diastole the heart is relaxed, when it seems more reasonable to trigger the shot. However, further research is needed to confirm this hypothesis. Since one study with competitive shooters was found, the evidence is insufficient to conclude that location of triggering cannot be used as a performance determinant in junior-level air-rifle shooters. Further studies are needed, especially with elite- and national-level air-rifle athletes, to classify and evaluate the existing findings with junior-level athletes.

Heart rate showed no influence on shooting performance in novice [27] and junior-level air-rifle athletes [28]. These results were confirmed with mixed groups of national-level small-bore athletes and novices [29], as well as with elite- and national-level small-bore shooters [13], where heart rate did not differ between superior and less successful shots. Results suggest that heart rate may not affect shot score. It could therefore be that there are certain ranges in which heart rate does not affect performance. Therefore, heart rate is not necessarily a performance determinant that can be used to discriminate between shot scores in novices and junior-level air-rifle shooters.

#### Small-bore shooting

In small-bore shooting, one study that compared groups found that elite-level athletes showed less upper body muscle activity than national-level shooters [29]. This could mean that upper body muscle activity may be used as a performance determinant to discriminate between elite- and national-level shooters.

Similarly, heart rate during the preparation period could differentiate between elite- and national-level small-bore athletes [13], but not between novices and national-level shooters [29]. In the last six seconds before shot release, elite-level athletes decreased their heart rate (from 87 to 84 beats per minute) less strong than national-level shooters (from 86 to 81 beats per minute). This may indicate that heart rate may be used as a performance determinant to discriminate between elite- and national-level shooters.

In addition, two studies showed that heart rate did not affect shot score in a mixed sample of national-level and novice small-bore athletes [29]; and, in elite- and national-level shooters [13]. These results were confirmed with novice [27] and national-level air-rifle athletes [28]. Here again, results may be interpreted as heart rate being independent of shot score. Since both studies in small-bore shooting did not control for skill level, the conclusiveness of the results is limited and only valid for the sample groups studied. However, results indicate that heart rate is not a useful performance determinant to discriminate between shot scores.

Further, a correlation was found between upper body muscle activity and rifle stability in a mixed sample of elite- and national-level small-bore shooters [29]. This means that lower muscle activity during the aiming and triggering process may lead to higher rifle stability. It is conceivable that the relationship between muscle activity and rifle stability plays a role for shooting performance with rifle stability being a mediator. However, since the authors of this study [29] did not control for skill level, the conclusiveness of the results is limited and only valid for the sample studied. So far, the direct influence of arm muscle activity on shot score has not been reported, thus, constituting a valuable further research target. However, there is evidence of improved shooting score after relaxation training in elite- and national-level biathletes [46]. Further, initial tendencies between low tension and high shooting score are provided in a study by Solberg et al. [8], who subjectively assessed the degree of tension on a visual analog scale (VAS). In national-level pistol shooters, muscle fatigue in the upper body was found in the last period of competition [47]. Further research could determine whether this also occurs in the rifle shooting and if muscle fatigue influences shooting performance.

#### Summary

No evidence-based conclusion can be drawn about the role of physiological parameters as performance determinants due to a lack of data. Initial results indicate that heart rate may not affect shooting performance in both air-rifle and small-bore shooting. Future research should re-examine the role of location of triggering within the cardiac cycle, as well as upper body muscle activity, to confirm or disprove whether these two factors can be used to distinguish between performance classes and/or shot scores. Care should be taken to ensure that elite- and national-level athletes are examined in future interventions and that the groups are analyzed separately. Since no studies exist on conditional abilities, it would be important to answer questions such as: how important is a good endurance level for shooters for long competition days? What is the influence of core muscles for the stability of the body, and therefore for rifle stability and shooting performance? Would a better strength in these muscle groups lead to improved shooting performance?

### Psychological factors

#### Air-rifle shooting

In air-rifle shooting, studies that compared groups found that highly skilled athletes showed lower state anxiety before competition compared to moderately skilled shooters, whereas no difference was found for trait anxiety and self-control [26]. These results are similar to Gulbinskienè & Skarbalius [48], who showed lower values for cognitive and somatic anxiety in high-level rifle shooters compared to moderate-level athletes. This may be interpreted as state anxiety being a performance determinant that may discriminate between elite- and national-level air-rifle athletes, while trait anxiety and self-control may not play a role in shooting performance.

In a mixed sample of high- and moderate-level air-rifle athletes, state anxiety correlated negatively with competition performance [26]: shooters with a lower level of state anxiety before competition showed better performance in the form of higher shot scores. Therefore, state anxiety may be used as a performance determinant to discriminate between shot scores in a mixed sample of high- and moderately-skilled air-rifle athletes. However, the time course remains unknown: since state anxiety fluctuates strongly across time, one could expect that athletes with better capability to control these anxiety states perform better. Sade et al. [26] reported weak correlations between trait anxiety, self-control and shot score, concluding that self-control and trait anxiety may not influence shooting performance.

#### Small-bore shooting

In small-bore shooting, lower stress levels led to higher shot scores in a mixed sample of national-level athletes and novices [30]. Normally, heart rate fluctuates across a small range and stressful situations may cause heart rate to exceed the upper limit of this range. This could mean that the change in heart rate due to stress is related to shooting performance. Stress level may therefore be used as a performance determinant to discriminate between shot scores. Stress could be one factor that explains, why shooters achieved worse results in competition compared to training [23]. One possible way to regulate pressure in competition is described in a study by Solberg et al. [8], who showed that meditation training led to an improved shooting performance.

#### Summary

No evidence-based conclusion can be drawn about the role of psychological factors as performance determinants due to lack of data. Initial trends exist regarding state anxiety as a performance determinant to distinguish between elite- and national-level air-rifle shooters and shot score. Further, stress may be used to discriminate between shot scores in national-level and novice small-bore shooters. Nevertheless, further research is needed to confirm the findings on state anxiety and stress both in air-rifle and small-bore shooting with elite- and national-level athletes. Here, it is important to use specific questionnaires in addition to physiological factors, such as heart rate, as a change in heart rate may have many reasons (e.g., endurance level, stress, anxiety). Further investigations could shed light on how other emotions fluctuate, especially in competition, and thereby affect shooting performance. Since it is not possible to assess these parameters with questionnaires during competition, other methods need to be developed. In addition to emotions, other shooting-related factors such as attention should be assessed in sport-related tests. Preliminary evidence is provided in a study by Doppelmayr et al. [49], who showed that elite-level rifle shooters increased their attention on the target at the time of triggering, compared to novices who maintained the same amount of attention.

### Comparison of performance determinants between air-rifle and small-bore shooting

The secondary objective of this review was to investigate whether the shooting performance of the two Olympic rifle disciplines are covered by the same performance determinants. However, the studies included in this systematic review focused on different performance determinants in air-rifle and small-bore shooting, therefore, this question cannot be answered (Table 2). Further, it became evident that small-bore shooting is far less investigated than air-rifle shooting. Nevertheless, some common performance determinants could be found for the two disciplines. First, rifle stability and body sway may be used as performance determinants to discriminate between elite- and national-level athletes. Second, although body sway may not influence shot score in elite- and national-level shooters, it could be a requirement to participate at a high level. Third, heart rate may not affect shot score in junior- and national-level athletes and therefore may not be an appropriate performance determinant to discriminate between shot scores in these athletes.

### Limitations

There’re several limitations of our review that need to be discussed. First, due to language barriers, we only included English and German articles in the study selection process, which may result in excluding performance determinants from articles written in other languages. However, most articles are written in English, so this should not be a major problem. Second, we excluded studies including EEG analyses or studies concerning drugs and nutrition intake. Therefore, it is not possible to make evidence-based conclusions about the influence of drugs and nutrition intake on shooting performance; instead we investigated performance determinants that can be easily trained. Third, the modified version of the Downs and Black quality assessment checklist needs to be validated in future research. However, modified versions have already been used in other studies in elite sports performance.

There’re also several limitations of the studies included in our review that need to be discussed. First, the definition of being an elite- or national-level athlete does not compare across countries. It is therefore possible that elite- and national-level athletes from different countries have the same mean shot score. We want to make readers aware of this difference in defining this categorization. Several studies also failed to state how the classification between elite- and national-level athletes was made. Second, different measuring systems and operationalization of the examined performance determinants were used, which may lead to differences in the methodological quality of studies and to difficulties in comparing studies. However, the same results were often seen regardless of the parametrization used. Third, other covariates exist that may have impacted the study results. For example, in some studies shooters did not use their own rifles, and, in some small-bore studies the distance to the target was reduced from 50 m to 18 m. In other studies, different numbers of shots or dry-firing shots were investigated. Fourth, some studies did not control for group affiliation, which limits the conclusiveness of the results to mixed-performance classes. Fifth, the analyses performed in some of the reviewed studies did not take into account the dependence of the measurements. For example, one study reviewed examined individuals’ parameters within a longitudinal design (i.e., each individual provided >300 data points). For this hierarchical data structure, more appropriate statistics should have been used, such as multi-level modeling.

## Conclusions and outlooks

To sum up, there is little research on performance-determining factors in air-rifle and small-bore shooting. It has been shown that in both types of shooting, horizontal and vertical rifle stability as well as body sway may be used as performance determinants to discriminate between elite- and national-level athletes. Since body sway did not influence shot score in elite-level air-rifle and small-bore athletes, it may be a performance requirement to compete at a high level. Further, in air-rifle shooting, horizontal rifle stability and aiming accuracy can be used as performance determinants to discriminate between shot scores on a national level. Inconsistent results, lack of data and methodological issues prevent us from drawing evidence-based conclusions on other performance factors.

The wide range of examined factors and possible influence on shooting performance also show that shooting performance is composed of multifaceted processes running simultaneously and sequentially, with possible independent contributions of distinct performance determinants. For example, body fluctuations affect rifle stability with a time delay, while holding ability and aiming accuracy run simultaneously in the aiming process and are therefore closely related to the subsequent triggering. For this reason, future research should focus not only on examining individual performance determinants but should also use more complex analyses comprising more than one performance determinant. Multiple regression analyses are already used, integrating several technical-coordinative factors concurrently into one model. Such analyses could help the understanding of how much single performance determinants contribute to shooting performance and how far performance can be predicted by several performance determinants. Evidence about the interaction beyond the edge of technical-coordinative parameters has not yet been made and should be considered for future research. Future studies could use structural equation models to uncover causality, as well as investigate the complex relationships between performance determinants and shooting performance.

Future research should focus on performance determinants from all categories to capture the multilayered influences on shot performance with athletes of different skill levels in both disciplines but especially with elite-level shooters. Psychological performance determinants should be given particular importance: although this area is underrepresented, it is an important component of sport shooting due to the high psychological requirements, such as concentration, that must be maintained during the whole competition for every shot. Future research should investigate possible performance determinants in small-bore shooting in the prone and kneeling positions to make a distinction between the three positions. Moreover, we call for studies with elite-level athletes from different countries and studies using high methodological quality to improve study outcomes. Research into performance determinants in the two Olympic rifle disciplines, air-rifle and small-bore shooting, is still in its infancy; but existing findings open avenues for promising future research endeavors.

## Supporting information

S1 Table PRISMA checklist. (DOC)

S2 Table Quality assessment. (DOCX)
