# Supplementary material for: Factors that influence performance in Olympic air-rifle and small-bore shooting: A systematic review
Source: PLoS One. 2021 Mar 31;16(3):e0247353. doi: 10.1371/journal.pone.0247353 (PMC8011779; doi:10.1371/journal.pone.0247353)
Supplement: S2 Table — (DOCX) [file pone.0247353.s002.docx]

S2 Table. Quality assessment of the studies reviewed using the Downs and Black quality assessment tool.

|  | Ball et al. (2003) | Era et al. (1996) | Ihalainen et al. (2016) | Ihalainen et al. (2016b) | Ihalainen et al. (2018) | Konttinen & Lyttinen (1992) | Konttinen et al. (1998) | Konttinen et al. (2003) | Kuitunen et al. (2013) | Landers & Qi (1985) | Mets et al. (2007) | Mon et al. (2019) | Sade et al. (1990) | Selva & Joseph (2017) |
| --- | --- | --- | --- | --- | --- | --- | --- | --- | --- | --- | --- | --- | --- | --- |
| Is the hypothesis/aim/objective of the study  clearly described? | 1 | 1 | 1 | 1 | 1 | 0.5 | 1 | 1 | 0.5 | 1 | 1 | 1 | 1 | 0.5 |
| Are the main outcomes to be measured clearly described in the Introduction or Methods section? | 1 | 0 | 1 | 1 | 1 | 0.5 | 1 | 1 | 1 | 0.5 | 0.5 | 1 | 1 | 1 |
| Are the characteristics of the subjects included in the study clearly described? | 0.5 | 0 | 0 | 0.5 | 0 | 0 | 0 | 0 | 0 | 1 | 0 | 1 | 0 | 0 |
| Are the interventions of interest clearly described? | 1 | 0.5 | 1 | 1 | 1 | 1 | 1 | 1 | 1 | 1 | 0.5 | 1 | 1 | 1 |
| Are the distributions of covariates in each group of subjects to be compared clearly described? | 1 | 1 | 1 | 2 | 1 | 1 | 1 | 2 | 1 | 1 | 1 | 1 | 1 | 0 |
| Are the main findings of the study clearly described? | 1 | 1 | 1 | 1 | 1 | 1 | 1 | 1 | 1 | 1 | 1 | 1 | 1 | 1 |
| Does the study provide estimates of the random variability in the data for the main outcomes? | 1 | 0.5 | 1 | 1 | 1 | 0 | 0.5 | 1 | 1 | 1 | 1 | 1 | 1 | 0 |
| Have all important adverse events that may be a consequence of the intervention been reported? | n/a | n/a | 0 | 0 | n/a | 0.5 | n/a | 1 | n/a | n/a | 1 | n/a | n/a | n/a |
| Have actual probability values been reported (e.g. 0.035 rather than <0.05) for the main outcomes except where the probability value is less than 0.001? | 1 | 0 | 0 | 0 | 0 | 0 | 0.5 | 0 | 0 | 0 | 0 | 0 | 1 | 0 |
| Were those subjects who were prepared to participate representative of the entire population from which they were recruited? | 0 | 0 | 0 | 0 | 0 | 0 | 0 | 0 | 0 | 0 | 0 | 1 | 0 | 0.5 |
| If any of the results of the study were based on  “data dredging”, was this made clear? | 1 | 1 | 1 | 1 | 1 | 0 | 1 | 0 | 1 | 1 | 0 | 1 | 1 | 1 |
| Were the statistical tests used to assess the main  outcomes appropriate? | 1 | 0 | 1 | 1 | 1 | 0 | 1 | 1 | 1 | 1 | 1 | 1 | 1 | n/a |
| Were the main outcome measures used  accurate (valid and reliable)? | 0 | 0 | 0 | 0 | 0 | 0 | 0 | 0 | 0 | 0 | 0 | 0 | 1 | 0 |
| Were study subjects of different groups tested in the same period of time? | n/a | n/a | 1 | 1 | 1 | n/a | n/a | n/a | 0 | n/a | n/a | n/a | 1 | n/a |
| Was there adequate adjustment for covariates in the analyses from which the main findings were drawn? | 0 | 0 | 0 | 0 | 1 | 0 | 0 | 0 | 0 | 0 | 1 | 0 | 0 | 0 |
| Did the study reported information about an a priori sample size calculation or a post-hoc power analysis? | 0 | 0 | 0 | 0 | 0 | 0 | 0 | 0 | 0 | 0 | 0 | 0 | 0 | 0 |

n/a = not applicable
